# SOCIAL PARTICIPATION AMONG OLDER ADULTS WITH UNMET NEED CONSEQUENCES: FROM A DISABILITY PERSPECTIVE

**DOI:** 10.1093/geroni/igad104.2353

**Published:** 2023-12-21

**Authors:** Natalie Turner, Brittany Jones

**Affiliations:** University of Washington, Seattle, Washington, United States; University of Washington, Seattle, Washington, United States

## Abstract

This study examines the relationship between social participation and consequences arising from unmet ADL, IADL, and mobility needs using disability studies frameworks. The aging field has yet to fully incorporate theoretical perspectives from disability scholarship into our understanding of health, wellbeing, accessibility and inclusion. By situating the experience of aging, aging with care needs, and having unmet care needs within a disability studies lens we gain insight into the ways we are called to redesign the provision of supportive services to ensure meaningful inclusion in community. To illustrate this idea, we use Wave 5 from the National Health and Aging Trends Study (NHATS) (n=7,070) to examine the relationship between ADL, IADL, and mobility unmet need consequences and social participation. Both social participation and unmet need consequences among older adults have been explored from healthcare, quality of life, and economic perspectives. However, there is a lack of scholarship linking these two concepts using a disability lens. Preliminary results from a series of linear regressions show a significant, negative relationship between presence of unmet ADL and mobility need consequences and social participation (p < .001). These findings indicate that experiencing unmet care needs may not only have detrimental effects on health, but on overall adult wellbeing and may be infringing on the ability of older adults to engage with community to the extent they wish. By applying disability frameworks, aging researchers are called to reexamine the ways in which we consider accessibility and full inclusion of older adults with care needs.

